# A Useful Invention

**Published:** 1883-04

**Authors:** 


*A Useful Invention.*—Dr. William Farmer, of Wytheville Va., has invented a mechanical appliance which will prove of great value to dental practitioners who practice outside of their regular offices. This invention consists of attachments which convert any ordinary chair into a convenient "dental chair," by elevating and adjusting it to suit the size of the patient and the locality of the work to be done in the mouth. The entire appliance is light, neat, and compact, capable of being packed in the space of a few inches square, and at the same time strong and durable. During commencement week, Dr. Farmer exhibited his appliance at the Infirmary of the Dental Department of the University of Maryland, where it was greatly admired for its convenience and usefulness.

---

### MONTHLY SUMMARY.

